# Localized amyloidosis presenting with a penile mass: a case report

**DOI:** 10.1186/1757-1626-2-160

**Published:** 2009-10-20

**Authors:** Joo Han Lim, Hoon Kim

**Affiliations:** 1 Department of Internal Medicine, Inha University Hospital, Shinheung-dong, Chung-gu, Incheon 400-711, Republic of Korea; 2 Department of Emergency medicine, Inha University Hospital, Shinheung-dong, Chung-gu, Incheon 400-711, Republic of Korea

## Abstract

Amyloidosis is a disease characterized by the deposition of altered proteins in tissues. Amyloid deposition always occurs in the extracellular matrix and presents a fibrillary conformation. Local deposition of amyloid may occur in individual organs, without systemic involvement. We report here a rare case of localized penile shaft amyloidosis--an unusual location for amyloid deposition--presenting as a penile mass that resulted in a urethral stricture in 37-year old male patient. We have also comprehensively reviewed the literature regarding localized amyloidosis.

## Introduction

Amyloidosis is a disease characterized by the deposition of altered proteins in tissues that shows the following characteristics: beta-pleated sheet molecular configuration with affinity to Congo red dye, fibrillar ultrastructure, and presence in the extracellular matrix leading to hardening of the affected tissues. Depending upon its extent, amyloidosis can be classified as systemic or localized disease. The reason for localized deposition is unknown, but it is hypothesized that such deposits result from the local synthesis of amyloid proteins rather than the deposition of light chains produced elsewhere. Isolated localized amyloidosis has been reported in almost every organ system. The localized form presents minor clinical manifestations as compared to the extensive organ involvement observed in systemic disease, and local amyloidosis may not be progressive [1]. Worldwide, there are few case report concerning localized amyloidosis of the penile shaft [2,3], and only a few reports of localized amyloidosis of the glans penis in Korea. We report here a rare case of localized amyloidosis of the penile shaft that manifested as urethral obstruction due to amyloid infiltration.

## Case presentation

A 37-year-old Asian male patient attended our clinic for evaluation of voiding difficulty and a tender mass at the distal urethra. His nationality is Korea. His chief complaint was discomfort on erection and during voiding. Physical examination revealed a tender mass in the penile shaft. His past, personal, and family history were unremarkable.

**Figure 1:**
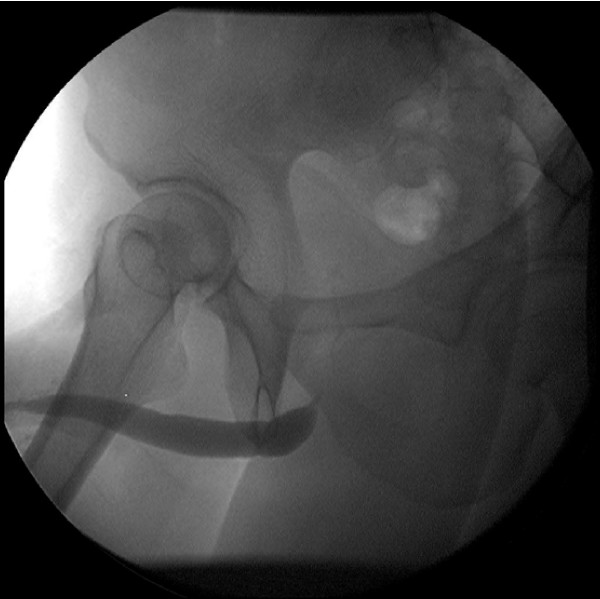
**RGP demonstrated a urethral stricture at the level of the bulbous urethra**.

**Figure 2:**
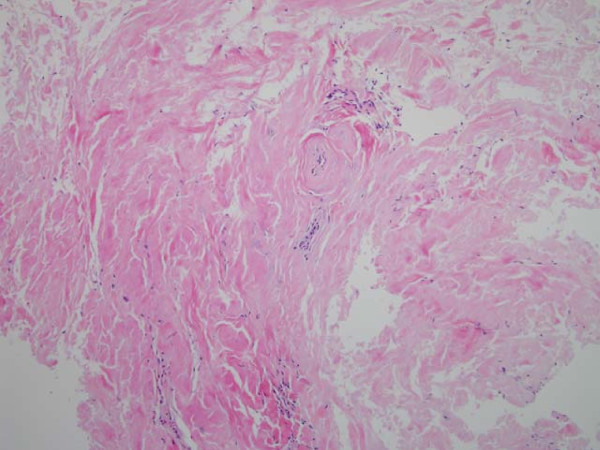
**Microscopic findings show amorphous eosinophilic amyloid deposition with Hematoxylin and Eosin stain**. (H&E, × 100).

**Figure 3:**
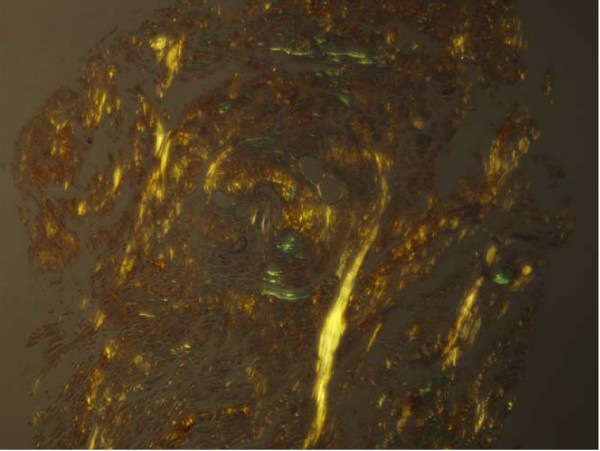
**The deposits exhibit apple-green birefringence under a polarized light microscope (Congo red stain)**.

Urinalysis was normal and urine cytology did not reveal any atypical cells. Blood cell count, blood biochemical investigation and urine culture were normal. Serologies for human immunodeficiency virus and syphilis were negative. A retrograde urethrogram (RGU) assess the presence of a urethral stricture; it revealed a stricture at the level of the bulbous urethra (Figure. 1). The patient underwent cystostomy for the severe urinary tract stricture. Further, tissue biopsy of the soft tissue mass of the penile shaft was performed. Histopathological examination revealed deposits of an amorphous, homogenous material (Figure. 2). Congo red staining was positive (Figure. 3). We subsequently performed a thorough work-up for evaluating the presence of systemic amyloidosis, including urine for Bence Jones proteins, serum proteins, serum immunoglobulin levels, free light chain assay, liver function tests, marrow examination, and rectal biopsy; all the test results were negative for systemic amyloidosis. After 1-year follow up, no changes in the clinical appearance have been observed; the patient has not developed any clinical or laboratory evidence of systemic amyloidosis.

## Discussion

The term amyloidosis refers to a group of disorders characterized by the extracellular accumulation of insoluble, fibrillar proteins in various organs and tissues [4]. Amyloidosis is classified as primary or secondary disease. In the primary type (AL amyloidosis), the amyloid deposit contains immunoglobulin light chains; generally, no etiology is apparent for the amyloid deposition. In the secondary type (AA amyloidosis), the amyloid deposit comprises serum protein A produced by the liver in response to cytokines produced due to complications of underlying chronic inflammatory diseases or multiple myeloma [5]. Amyloidosis can also be classified according to its anatomic placement as localized disease (10-20%) or systemic disease (80-90%) [6]. Most cases of amyloidosis occur as systemic disease, and have a poor prognosis. It is important to distinguish primary localized amyloidosis from systemic amyloidosis because of their divergent prognoses. Systemic amyloidosis is frequently distinguished from localized amyloidosis by demonstrating protein in subcutaneous fat, rectal mucosa, bone marrow, urine, or serum. Localized amyloidosis is diagnosed primarily by exclusion of the above. The lack of myelomatous, infectious, familial, or other systemic diseases, as well as negative results on the respective specific immunostains, excludes the more common systemic amyloidoses [7]. Localized amyloidosis has been described in nearly every organ system including lung, larynx, skin, tongue, eye, and genitourinary tracts [1,6,8]. Localized amyloidosis of the penile shaft is a very rare disease entity. Progression from localized amyloidosis to systemic amyloidosis is known to be very uncommon [9]. Specific treatment is available for every type of amyloidosis. A combination of prednisone/mephalan/colchicines has proven effective. But in reported case of localized amyloidosis, local excision was the rule. Outcome was generally favorourable.

In our case, the patient did not exhibit any clinical or laboratory evidence of systemic disease, suggesting the local production of amyloid protein. Moreover, as occurred in our case, localized amyloidosis in the penile area should be considered among the differential diagnoses of urinary tract obstruction.

In conclusion, we have reported here a very rare case of localized amyloidosis presenting as a penile mass that manifested as a urinary tract stricture and that was confirmed by tissue biopsy. To the best of our knowledge, this is the sixth case of genitourinary tract-localized amyloidosis and the first case in Korea presenting with a penile mass that resulted in a urinary tract stricture. A consideration of localized amyloidosis of penile area should be considered in cause unknown urinary obstruction cases. Further study of localized amyloidosis may be warranted for elucidating the underlying pathophysiology of localized amyloidogenesis, which could provide a greater understanding of this disease as well as point toward its treatment.

## Consent

Written informed consent was obtained from the patient for publication of this case report and accompanying images. A copy of the written consent is available for review by the Editor-in-Chief of this journal.

## Competing interests

The authors declare that they have no competing interests.

## Authors' contributions

HK analyzed and interpreted the patient data regarding the hematological disease. JHL was a major contributor in writing the manuscript. All authors read and approved the final manuscript
